# Mesial temporal lobe epilepsy: A West Indian Neurosurgical Experience

**DOI:** 10.1016/j.ijscr.2019.10.063

**Published:** 2019-11-04

**Authors:** Avidesh Panday, Chrystal Calderon, Sherry Sandy, Devindra Ramnarine

**Affiliations:** a Department of Medicine, Eric Williams Medical Sciences Complex (NCRHA), Trinidad and Tobago; b Department of Surgery, Eric Williams Medical Sciences Complex (NCRHA), Trinidad and Tobago; c Department of Medicine, Port of Spain General Hospital (NWRHA), Trinidad and Tobago

**Keywords:** Mesial temporal lobe epilepsy, Medical refractory epilepsy, Anterior temporal lobectomy, Caribbean, Case report

## Abstract

**Introduction:**

We sought to highlight a case of refractory mesial temporal lobe epilepsy managed via an anterior temporal lobectomy. To our knowledge, this is the first of its kind to be performed and published in the English-speaking Caribbean.

**Presentation of case:**

A 44-year-old female, known seizure disorder for 25 years was referred to the outpatient clinic. Several trials of varying anti-epileptic drugs failed to provide remittance of her frequent partial seizures over the last two decades.

Magnetic resonance imaging displayed asymmetry of the hippocampus, with a prominent right temporal horn of lateral ventricle, in keeping with right anteromesial temporal lobe sclerosis. She underwent a right anterior temporal lobectomy after exhaustive counselling; with the guidance of a multidisciplinary team. The post-operative period was uneventful and currently, the patient has been seizure-free since operation.

**Discussion:**

Drug resistant epilepsy is defined as failure of adequate trials of two tolerated, appropriately chosen and used anti-epileptic drug schedules to attain sustained seizure freedom. Medical management of mesial temporal lobe epilepsy has a relatively poor long-term outcome, with 30 % of patients falling into the category of pharmaco-resistant.

**Conclusion:**

Surgical management of these cases have been proven to be a safe and effective alternative but is currently greatly underutilized. In our literature review, we present the first published case of epilepsy surgery in the English-speaking Caribbean for temporal lobe epilepsy.

## Highlights

• Mesial temporal lobe epilepsy represents a large proportion of medical refractory or drug-resistant epileptic cases.
• Greater initiatives are mandated for earlier assessment of suitable surgical cases.
• The combined efforts of multiple disciplines are essential to attaining progress in this endeavour.
• Surgical intervention provides seizure freedom and significant improvement in quality of life.
• The first published case of successful epilepsy surgery in the English-speaking Caribbean for temporal lobe epilepsy.

## 1 Introduction

Epilepsy affects all aspects of a patient’s life. Independent of seizure-related mortality, there are neurocognitive and stigma-related ramifications. In Trinidad and Tobago, the Pan American Health Organisation (PAHO) estimates that approximately 40,000 persons are living with epilepsy. Notably, it was stated that the mortality from epilepsy seen in Latin America and the Caribbean is 1.04 per 100,000 population, comparatively higher than what is seen in more developed countries [1,2].

Despite the use of anti-epileptic medications, many patients will continue to have seizures – along with the additional exposure to its side effect profile [3]. In the first world, epilepsy surgery has progressed over the past 20 years where many drug-resistant patients are identified early on and offered surgical evaluation. Mesial temporal lobe epilepsy (MTLE) is a major contributor to the group of medically refractory epilepsies. It is amenable to surgical management and is one of the leading indications for epilepsy surgery [4].

We present a case of drug-resistant MTLE, the patient was offered neurosurgical evaluation and went on to have successful surgery in a public tertiary heath facility. It is on this backdrop that we hope this will promote drug-resistant epilepsy awareness and the need for the development of a comprehensive Epilepsy Program in Trinidad and Tobago.

This article has been completed in accordance to the SCARE criteria [5].

## 2 Presentation of case

We present the case of a 44-year-old right-handed female with a 25-year history of epilepsy.

Her seizures were of multiple subtypes:

1) Focal impaired awareness with cognitive changes and behavioural arrest: the patient would experience intense déjà vu just prior to seizure onset and on some occasions may have déjà vu alone.
2) Focal impaired awareness with cognitive changes and behavioural arrest progressing to bilateral tonic-clonic seizures.

Overall, her seizure frequency was variable, ranging from a minimum of 2 per month to a maximum of 8 per month.

Her epilepsy risk factors were significant – with a history of febrile seizures as a baby. Her major precipitating factors for seizures were related to high stress situations and hormonal changes.

The patient had been previously trialled on various anti-epileptic drugs (AEDs) inclusive of carbamazepine, levetiracetam, lacosamide, clonazepam and lamotrigine. During a 25-year spell the patient had been on various combinations of these drugs. Furthermore, on multiple occasions she was exposed to treatment with at least three different AEDs combinations. Her medical co-morbidities included anxiety and a history of memory deficits, which substantially affected the occupational aspects of her life.

**Fig. A1:**
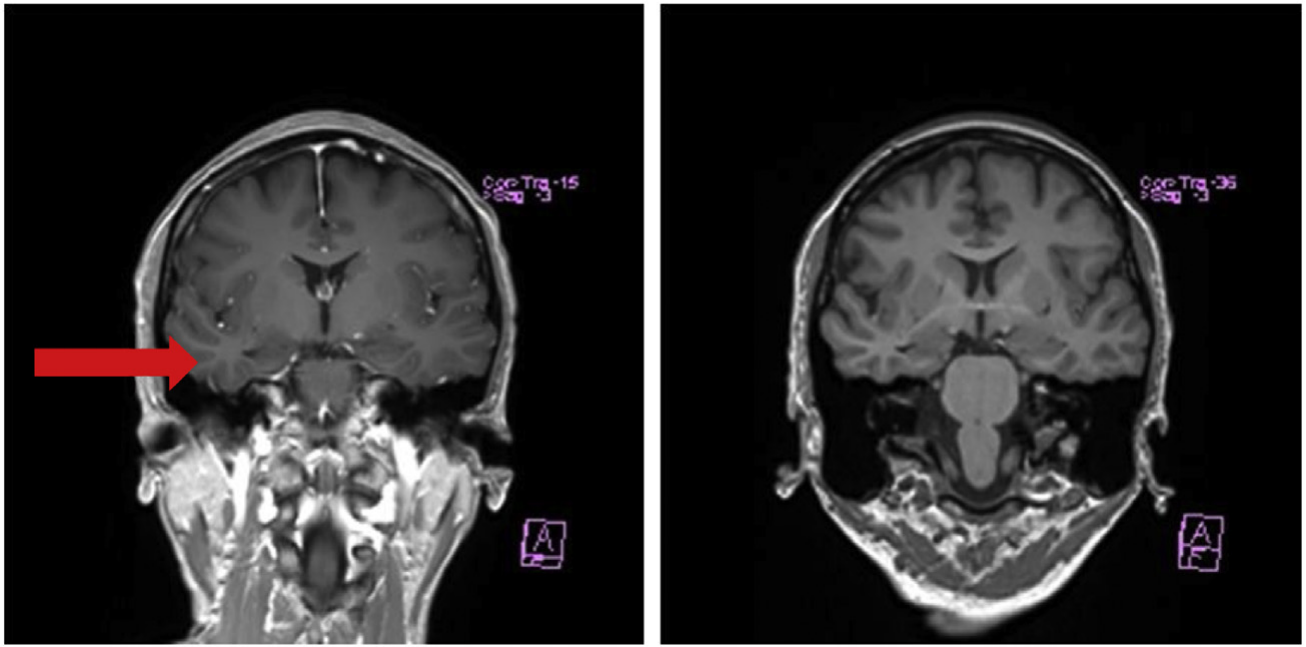
MRI Brain (coronal views) showing asymmetry of the lateral ventricles, with a prominent right temporal horn of the lateral ventricle, indicative of decreased volume in the right hippocampal region.

Medical records over her entire history were difficult to obtain due to dissemination across multiple facilities. However, when the patient presented for an Epilepsy Review at our service, it was clear that she fulfilled the definition of medically refractory epilepsy. She was referred for a 72-h ambulatory electroencephalogram (EEG) and 3T magnetic resonance imaging (MRI). This EEG revealed focal slowing in the form of right Temporal Intermittent Rhythmic Delta Activity (TIRDA) with rare epileptiform potentials in the right anterior temporal region. Her 3T MRI revealed evidence of marked right mesial temporal lobe sclerosis (Fig. A1). Neuropsychological assessment followed, which demonstrated evidence of mild neurocognitive deficits in the realm of verbal memory.

**Fig. A2:**
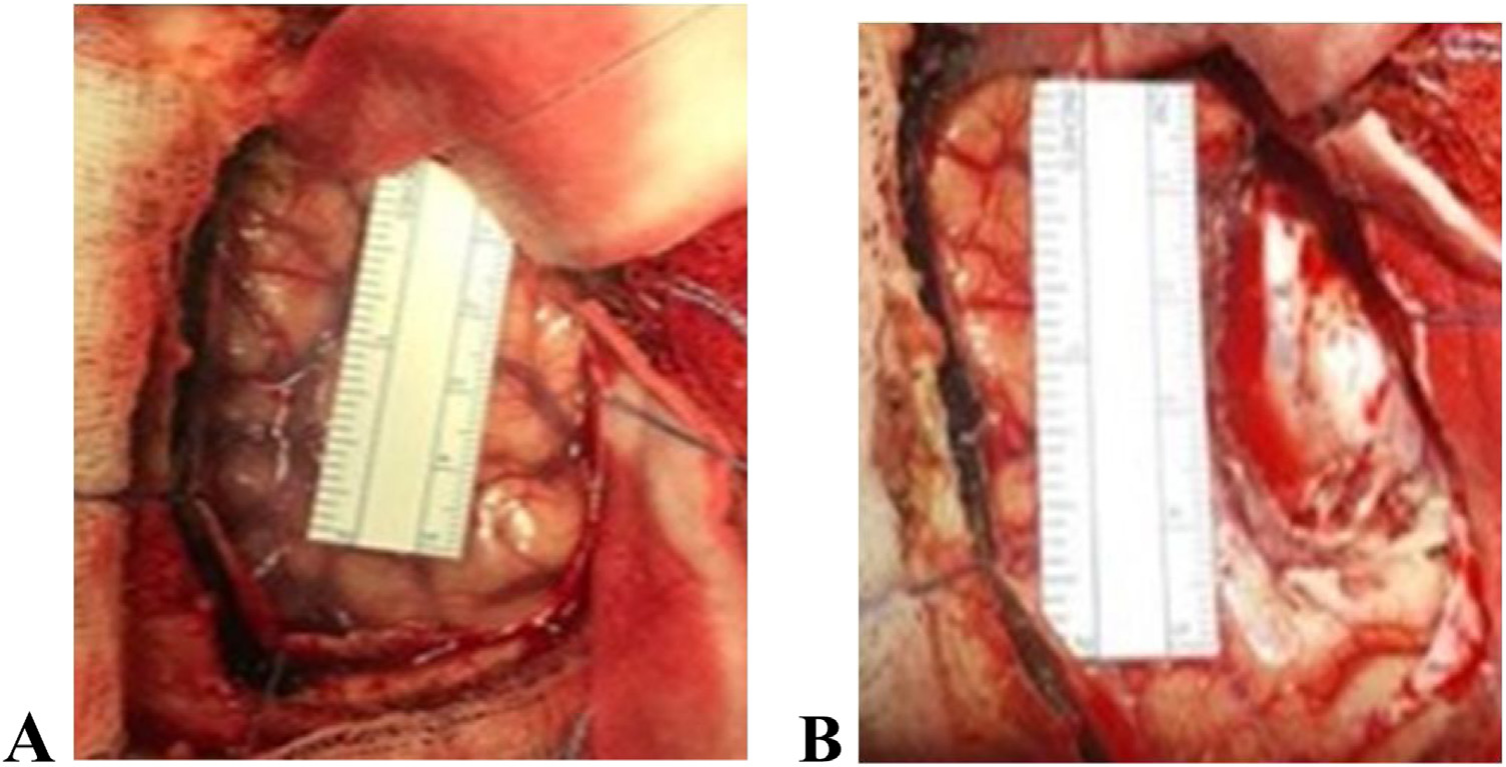
Intra-operative pictures showing the extent of neocortical dissection A. before neocortical resection and B. after neocortical resection with exposure medial temporal lobe structures.

At the inaugural Epilepsy Case Conference in 2018, it was decided that the patient would benefit from a standard right Anterior Temporal Lobectomy performed by the senior neurosurgery consultant. Surgical intervention took place in August 2018 in the public health setting (Fig. A2). As of the time of submission (June 2019), the patient has had no further seizures and continues to be on AEDs. She is pleased with this new seizure-free lifestyle, which was once thought to be unattainable. A repeat routine EEG done in February 2019 was within normal limits. On reaching the one-year mark post-surgical intervention, a decision will be made on medication weaning.

## 3 Discussion

AMTLE is one of the common causes of medical refractory seizures in adults and its focus stems from the hippocampus [6]. The hippocampus is part of the archicortex of the brain and has been classically linked to the limbic system, and hence its importance to memory. Memory impairment is a common complaint in temporal lobe epilepsy, as seen in our case patient. This was a significant aspect of her disability, as it directly affected her productivity in the work and home environment. This element of cognitive impairment adds a significant facet to an already disabling condition and most of these patients will benefit from neuropsychological intervention in their management.

Other key findings in their semiology include the presence of auras (altered somatic sensation or emotions) followed by various forms of automatisms. Our patient’s aura was déjà vu and is the first recollection of focal seizure without loss of awareness. These seizures may progress to loss of awareness, usually taking the form of behavioural arrest or staring [7]. Other persons may exhibit lip smacking, picking at clothes or speaking unintelligibly.

With the addition of features deduced from semiology, a diagnosis of mesial temporal lobe epilepsy is based on a consortium of investigative techniques: interictal scalp EEG, video – EEG and numerous imaging techniques – typically MRI. Interictal scalp EEG shows intermittent slow waves at 1–3 Hz (TIRDA) or 4–7 Hz (theta activity) from the ipsilateral or predominantly from pathologic temporal lobe. Ictal EEG shows the classic finding of anterior temporal spikes, often proceeded by a slow wave [8,9]. High resolution MRI is highly sensitive in its role of detecting structural abnormalities in the temporal lobe. Imaging scans depict volumetric asymmetry of the affected hippocampus, hyperintense signal on T2 weighted imaging/FLAIR (fluid- attenuated inversion recovery), loss of definition of the internal structures of the hippocampus, and asymmetry of the temporal horns of the lateral ventricle [10,11]. Other imaging tools that may play a critical role in surgical planning include functional MRI, diffusion tensor imaging and single-photon emission computed tomography (SPECT) [8,10].

Medical management of mesial temporal lobe epilepsy has a relatively poor long-term outcome, with 30 % of patients falling into the category of pharmaco-resistant. Polytherapy was attempted in this case patient but failed to demonstrate the desired clinical effect. Drug resistant epilepsy is defined as failure of adequate trials of two tolerated, appropriately chosen and used antiepileptic drug schedules to attain sustained seizure freedom [12]. The underlying mechanisms used to describe this failed pharmacotherapy relates to the effect and transportation of the drug, and possible changed brain homeostasis [13]. Fortunately, surgical intervention has been shown to be superior to medical therapy, with improvements in quality of life and attenuation in the likelihood of adverse events of this condition [14].

The two commonly used surgical techniques for mesial temporal lobe epilepsy are an anterior temporal lobectomy and the selective amygdalohippocampectomy (SAH) [15]. In this case patient we employed an anterior temporal lobectomy with resection of the hippocampus, similar to what was classically described by Spencer et al. [16]. Overall, surgical management of mesial temporal lobe epilepsy provides seizure freedom in 85 % of patients at 2-year follow- up [14]. These results and many others like it have led to greater advocacy for surgical intervention to be part of routine case management much earlier than what is currently noted.

## 4 Conclusion

Surgical intervention for mesial temporal lobe epilepsy is one of the most common and successful procedures performed globally. This is the premier drug-resistant epilepsy case managed via neurosurgical intervention, and published in the English-speaking Caribbean, based on our literature search. It required the collaborative efforts of neurology, neurosurgery and radiology. This further highlights the need for the establishment of a neuroscience multi-disciplinary team in tertiary centres across the Caribbean. More work is required to attain thorough investigative studies for these patients, but through the spread of information this can be achieved. The work done here represents a promising start.

## Sources of funding

None to be stated.

## Ethical approval

Ethical approval is exempt in this case publication.

## Consent

Patient consent was obtained for the publication of this case.

## Author’s contribution

Dr Avidesh Panday: conceptualisation, validation, investigation, writing - original draft, writing - review and editing, project administration.

Mr Devindra Ramnarine: conceptualisation, validation, investigation, resources, writing - review and editing.

Dr Sherry Sandy: conceptualisation, validation, writing- original draft, writing - review and editing.

Dr Chrystal Calderon: conceptualisation, writing- original draft, writing - review and editing, visualization.

## Registration of research studies

This is not applicable in this case publication.

## Guarantor

Dr Avidesh Panday - Consultant Physician, FRCP (Edin).

Mr Devindra Ramnarine - Consultant Neurosurgeon, MBBS, FRCS Ed (Neuro. Surg.).

## Provenance and peer review

Not commissioned, externally peer-reviewed.

## Declaration of Competing Interest

None to be stated.

## Figures and Tables

**Fig. A3:**
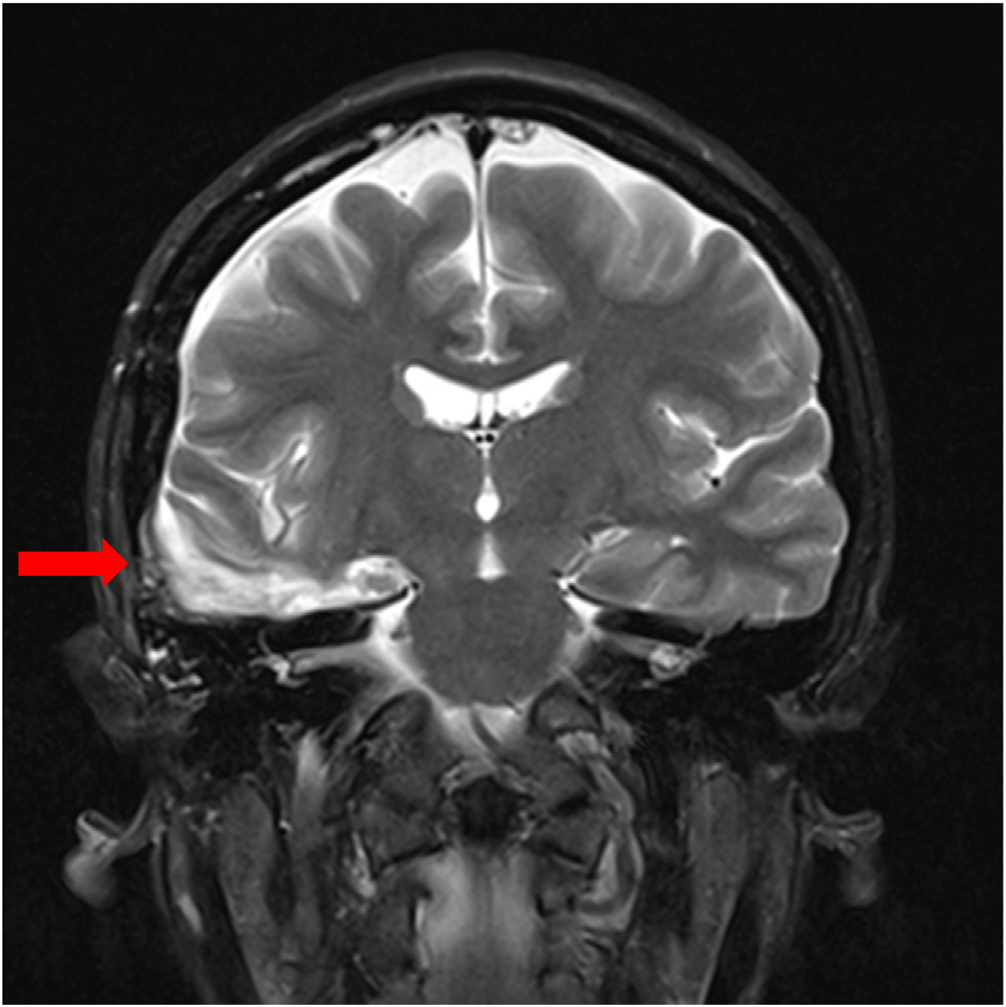
MRI Brain T2 weighted (coronal view) demonstrating post-surgical changes following right anterior temporal lobectomy.

## Appendix A

See Fig. A3.
